# The predictive value of changes in left atrial volume index for rehospitalization in heart failure with preserved ejection fraction

**DOI:** 10.1002/clc.23952

**Published:** 2022-11-20

**Authors:** Zhujing Hao, Guiwen Xu, Mengyang Yuan, Yuxi Sun, Ruopeng Tan, Yang Liu, Yun‐Long Xia

**Affiliations:** ^1^ Institute of Cardiovascular Diseases The First Affiliated Hospital of Dalian Medical University Dalian China; ^2^ Department of Cardiology, Institute of Heart and Vascular Diseases The First Affiliated Hospital of Dalian Medical University Dalian China

**Keywords:** heart failure with preserved ejection fraction, left atrial volume index, rehospitalization

## Abstract

**Aims:**

Left atrial volume index (LAVI) is an adequate analysis to predicate the left ventricle (LV) filling pressures, providing a powerful predictive marker of LV diastolic dysfunction. LAVI is a dynamic morphophysiological marker, and whether LAVI changes can predicate clinical outcomes in HF with preserved ejection fraction (HFpEF) is unknown.

**Methods:**

HFpEF patients were retrospectively studied from the First Affiliated Hospital of Dalian Medical University. Patients were classified into deteriorated, stable and improved groups according to the change in LAVI. Rehospitalization was defined as the main endpoint, the composite outcome of rehospitalization or all‐cause death was defined as the secondary endpoint.

**Results:**

A total of 409 patients were included. In this cohort, the percentage of deteriorated, stable, and improved LAVI were 99 (24.2%), 235 (57.4%), and 75 (18.4%), respectively. During the 22 months follow‐up period, 168 patients (41.1%) were rehospitalized, 31 patients (7.5%) died and 182 patients (44.5%) experienced a composite outcome. Multivariate Cox regression showed that compared to improved LAVI, those with deteriorated and stable LAVI experienced higher risk of rehospitalization. Logistic regression showed atrial fibrillation (AF) and higher creatinine were independent predictors of deteriorated LAVI, whereas the use of loop diuretics, calcium channel blockers (CCB), and high level of high‐density lipoprotein cholesterol (HDL‐C) were significantly associated with improved LAVI.

**Conclusions:**

Change in LAVI provides a powerful and dynamic morphophysiological marker of LV filling status and can be used to evaluate the rehospitalization in HFpEF patients.

## 1 INTRODUCTION

Heart failure (HF) is a leading cause of mortality and morbidity worldwide and affects at least 26 million people.^1^ According to the left ventricular ejection fraction, HF was classified into HF with reduced ejection fraction (HFrEF), HF with mid‐range ejection fraction (HFmrEF), and HF with preserved ejection fraction (HFpEF). Among them, HFpEF has grown to become the dominant form of HF, and increased with age, reaching 70% in patients >65 years of age.^2^, ^3^, ^4^ To date, no treatment has been shown to convincingly reduce mortality and morbidity in patients with HFpEF.^5^ Thus, it is urgent to search for a reliable marker which can effectively predict the prognosis of HFpEF.

HFpEF is characterized by left ventricular (LV) hypertrophy, LV diastolic dysfunction, and left atrial (LA) enlargement.^6^ Left atrial volume index (LAVI), measured by two‐dimensional (2D) echocardiography, is an accurate descriptor of LA volume, and is recommended in the latest guidelines for the diagnosis of HFpEF.^5^ The 2015 recommendations for cardiac chamber quantification by echocardiography in adults from American Society of Echocardiography (ASE) in association with the European Association of Cardiovascular Imaging (EACVI) indicated that LAVI can be categorized as normal (≤34 ml/m^2^), mild dysfunction (34–41 ml/m^2^), moderate dysfunction (42–48 ml/m^2^), and severe dysfunction (>48 ml/m^2^).^7^ However, LAVI is a dynamic parameter, the expansion of LAVI occurred during cardiac injury, abnormal neurohormonal factors, sustained cardiac pressure and/or volume overload.^8^ In contrast, such expansion could be reversed by eliminating the risk factors or receiving effective treatment.^9^ At present, most previous studies focus solely on detecting the associations between baseline LAVI and clinical outcomes, with few attentions paid to the prognostic value of LAVI alteration for HFpEF.

## 2 METHODS

### 2.1 Study population and group

We conducted a single‐center retrospective cohort study enrolled patients with a diagnosis of HFpEF who were hospitalized at the First Affiliated Hospital of Dalian Medical University, between 1 January 2018 and 30 June 2020. Baseline characteristics, including comorbidities, drug therapy, laboratory values, and echocardiographic parameters were obtained during the first hospitalization. The second echocardiogram was performed at the time between 6 and 12 months follow‐up in outpatient. It's worth noting that as there is no convincingly effective treatment recommended for HFpEF, all enrolled patients received individualized medical therapy according to disease severity, clinical characteristics and comorbidities.

**Figure 1:**
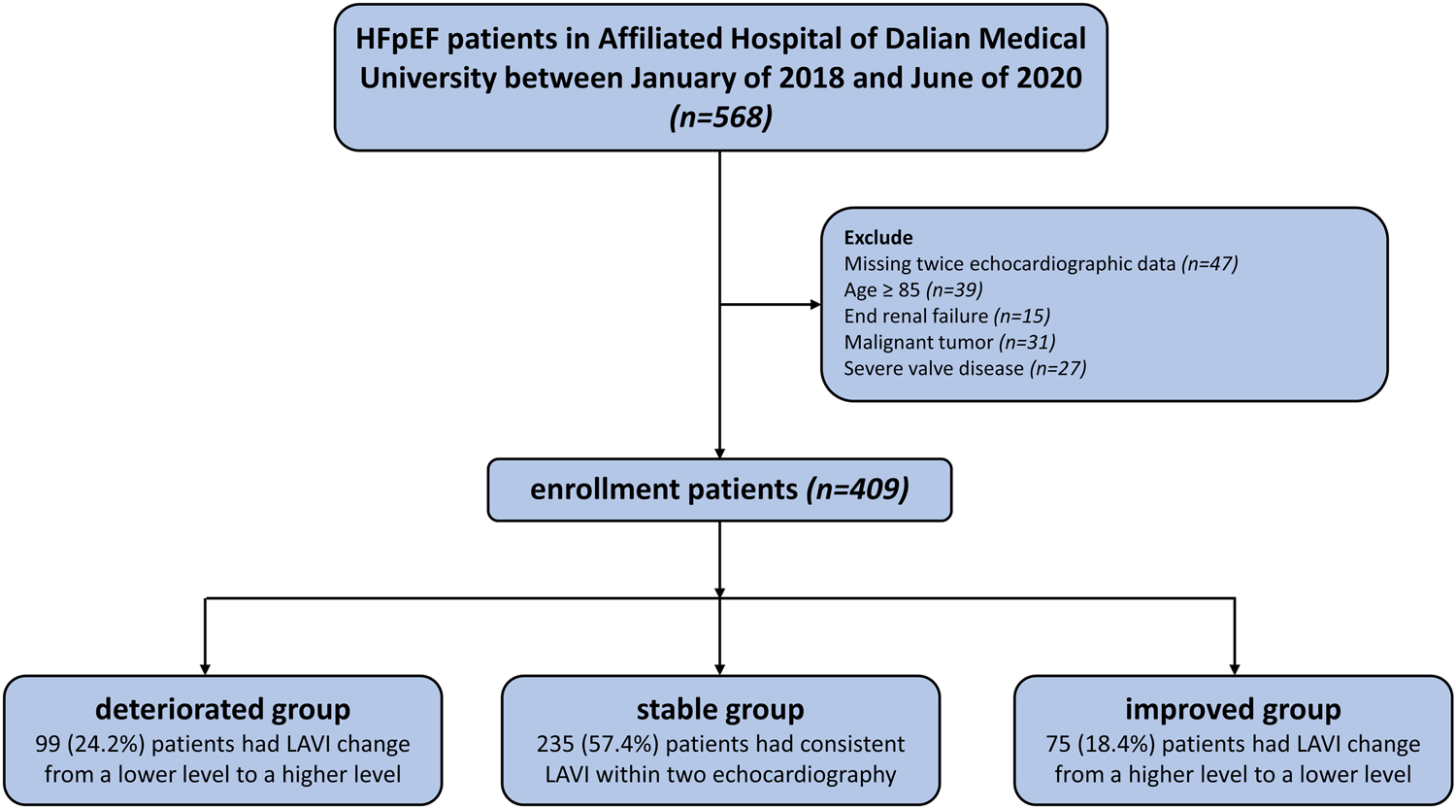
Flow diagram of patient identification, exclusion, and classification

The LAVI values measured by echocardiography were categorized into four different levels: normal (≤34 ml/m^2^), mild dysfunction (34–41 ml/m^2^), moderate dysfunction (42–48 ml/m^2^), and severe dysfunction (>48 ml/m^2^). Patients were grouped based on the degree of LAVI variation between the two echocardiography measurements and the grouping principles are as follows: (1) deteriorated group was defined as LAVI changed from a low level to a high level; (2) stable group was defined as LAVI maintained within the same level. (3) improved group was defined as LAVI changed from worsened to a better level. Patients over 85 years were excluded due to the higher risk of comorbid conditions (*n* = 39). Other exclusion criteria for all participants included missing twice echocardiographic data (*n* = 47), significant renal dysfunction (estimated glomerular filtration rate <30 ml/min per 1.73 m^2^) (*n* = 15), malignant tumor (*n* = 31), moderate or severe valve disease (*n* = 27) (Figure 1). The study was approved by the ethics committee of the First Affiliated Hospital of Dalian Medical University.

### 2.2 Clinical definition

HFpEF was diagnosed according to the 2021 ESC guidelines for the diagnosis and treatment of acute and chronic HF.^5^ The diagnostic criteria are as following: (1) symptoms and signs of HF, (2) LVEF ≥ 50%, (3) objective evidence of cardiac structural and/or functional abnormalities consistent with the presence of LV diastolic dysfunction/raised LV filling pressures, including raised natriuretic peptides (B‐type natriuretic peptide (BNP) >35 pg/ml or N‐terminal pro B‐type natriuretic peptide (NT‐proBNP) >125 pg/ml).

### 2.3 Echocardiographic measurements

Echocardiography examinations were carried out by using a Vivid 7 ultrasound system with an M5S transducer (1.7–3.3 MHz) (Vingmed Ultrasound, GE). All images acquired were stored digitally for off‐line analysis (EchoPAC version 202, Vingmed Ultrasound, GE). LA maximal volume was obtained from apical four‐chamber and two‐chamber views at end‐systole through the modified Simpson disc method, and then normalized to body surface area (BSA) to derive LAVI. Other echocardiography measures were assessed according to ASE guidelines by experienced radiologists.

### 2.4 Endpoints and follow‐up

The primary endpoint of this study was the occurrence of rehospitalization, and the secondary endpoint was the composite outcome of rehospitalization or all‐cause death. The follow‐up duration was calculated from last echocardiography to the occurrence of main endpoints or until 30 June 2021. All patients were encouraged to return to our outpatient clinic at least every 2 months. Telephone contact was maintained with the patients who were absent for their regular outpatient clinic.

### 2.5 Statistical analysis

Continuous variables with normal distribution were presented as the mean ± standard deviation, and nonparametric variables were expressed as the median and interquartile range. Categorical variables were expressed as numbers and percentages. Comparison of baseline characteristics among multi‐groups was performed using *χ* ^2^ test for qualitative variables and the ANOVA test or Wilcoxon rank‐sum test for the quantitative variable. The correlation between clinical variables and LAVI alterations was assessed by univariate and multivariate logistic regression analysis. The predictors with *p* < .05 in the univariate analysis were included in the multivariable model. Odds ratio (OR) and 95% confidence interval (CI) were obtained. Univariate and multivariate Cox proportional hazards regression models were constructed to detect the association between risk factors and adverse outcomes. All predictors in the univariate analysis with a significance of *p* < .05 were entered into the multivariable model. Hazard ratio (HR) and corresponding 95% CI were presented. Freedom from rehospitalization, all‐cause death and composite outcome in the cohort were analyzed with cumulative incidence curve Kaplan–Meier analysis and compared differences with the log‐rank test. Two‐sided *p* < .05 were considered statistically significant. Statistical analyses were performed using Statistical Package for Social Sciences, Version 26.0 (SPSS Inc) and R software.

## 3 RESULTS

### 3.1 Baseline characteristics

Of the 568 patients initially included, 159 were excluded due to meet preset exclusion criteria, resulting in a total of 409 patients were ultimately enrolled in this study. In the cohort, 99 (24.2%) were assigned into the deteriorated group, 235 (57.4%) were assigned into the stable group, and 75 (18.4%) were assigned into the improved group (Figure 1).

The baseline clinical characteristics were shown in Table 1. In the cohort, patients in stable group had the lowest incidence of atrial fibrillation (AF) among three groups, while the rate was comparable between deteriorated group and improved group. In terms of drug therapy, patients in stable group were less likely to receive loop diuretics and spironolactone than improved group. In addition, patients in improved group were more likely to take calcium channel blocker (CCB) than another two groups. In terms of laboratory values, patients in the stable group had lowest level of high‐sensitivity troponin (hs‐TnI), whereas those in improved group had highest concentration of high‐density lipoprotein cholesterol (HDL‐C) among three groups. As for echocardiographic indicators, the stable group had lower value of left ventricular posterior wall thickness (LVPWT) than deteriorated and improved groups, whereas the improved group had the highest baseline LAVI.

**Table 1:** Baseline demographics and clinical characteristics of patients at the time of first echocardiogram

Variables	Total population	Deteriorated group	Stable group	Improved group	*p* Value
Case（*n*, %)	409 (100)	99 (24.2)	235 (57.4)	75 (18.4)	‐
Age (years)	70.3 ± 11.5	72.2 ± 10.2	69.4 ± 12.0	70.5 ± 11.2	.119
Female	255 (62.3)	61 (61.6)	144 (61.3)	50 (66.7)	.693
BMI	26.0 ± 3.9	26.1 ± 3.3	25.8 ± 4.2	26.5 ± 4.1	.455
NYHA					
II (*n*, %)	68 (16.6)	18 (18.2)	37 (15.7)	13 (17.3)	.864
III (*n*, %)	275 (67.2)	66 (66.7)	159 (67.7)	50 (66.7)	.981
IV (*n*, %)	66 (16.1)	15 (15.1)	39 (16.6)	12 (16.0)	.988
Systolic blood pressure (mmHg)	138.9 ± 25.0	134.3 ± 23.5	139.2 ± 24.1	143.7 ± 29.0*	.049
Diastolic blood pressure (mmHg)	77.9 ± 13.9	77.7 ± 12.3	78.4 ± 13.5	76.9 ± 16.9	.717
Atrial fibrillation (*n*, %)	228 (55.7)	69 (69.7)	113 (48.1)*	46 (61.3)**	.001
Hypertension (*n*, %)	280 (68.5)	65 (65.7)	156 (66.4)	59 (78.7)	.108
Diabetes (*n*, %)	137 (33.5)	34 (34.3)	71 (30.2)	32 (42.7)	.135
PCI (*n*, %)	57 (13.9)	13 (13.1)	36 (15.3)	8 (10.7)	.578
Pacemakers (*n*, %)	46 (11.2)	15 (15.2)	22 (9.4)	9 (12)	.302
Drug therapy					
Loop diuretics (*n*, %)	277 (67.7)	71 (71.7)	144 (61.3)	62 (82.7)**	.002
βeta‐blockers (*n*, %)	328 (80.2)	78 (78.8)	183 (77.9)	67 (89.3)	.088
ACEI/ARB (*n*, %)	225 (55.0)	51 (51.5)	127 (54.0)	47 (62.7)	.308
Spironolactone (*n*, %)	220 (53.8)	56 (56.6)	115 (48.9)	49 (65.3)**	.038
CCB (*n*, %)	205 (50.1)	45 (45.5)	109 (46.4)	51 (68.0)*,**	.003
Digoxin (*n*, %)	58 (14.2)	14 (14.1)	34 (14.5)	10 (13.3)	.970
Statin (*n*, %)	245 (59.9)	63 (63.6)	135 (57.4)	47 (62.7)	.496
Antiplatelet drugs (*n*, %)	186 (45.5)	47 (47.5)	102 (43.4)	37 (49.3)	.602
Laboratory values					
Hemoglobin (g/ml)	124.5 ± 23.7	121.8 ± 25.9	125.3 ± 24.1	125.2 ± 19.0	.444
d‐dimer	390.0 (220.0, 803.0)	340.0 (182.5, 677.5)	410.0 (210.0, 922.5)	390.0 (240.0, 847.5)	.286
BNP (pg/ml)	231.76 (133.8, 458.2)	222.7 (139.8, 513.4)	230.12 (105.5, 411.4)	275.3 (161.2, 471.6)	.247
NT‐proBNP (pg/ml)	981.3 (449.5, 1953.5)	1056.5 (578.3, 2193.0)	949.9 (343.2, 2017.9)	1105.0 (579.45, 1589.9)	.672
hs‐TnI (ug/L)	0.02 (0.01, 0.04)	0.02 (0.01,0.05)	0.01 (0.01,0.03)*	0.02 (0.01,0.04)**	.004
Creatinine (mmol/L)	75.0 (60.0, 93.5)	76.0 (61.0, 101.0)	74 (59.0, 92.0)	76.0 (63.0, 99.0)	.509
Urea (mmol/L)	7.2 (5.4, 9.4)	7.27 (5.7, 10.5)	7.1 (5.4, 9.1)	7.6 (5.4, 9.5)	.314
UA(mmol/L)	371 (285, 474.5)	391 (281, 485)	370 (288, 469)	346 (276, 481)	.424
TC (mmol/L)	4.3 ± 1.2	4.3 ± 1.2	4.2 ± 1.1	4.4 ± 1.2	.418
TG (mmol/L)	1.1 (0.8, 1.7)	1.1 (0.8, 1.7)	1.1 (0.8, 1.6)	1.1 (0.9,1.7)	.555
HDL‐C (mmol/L)	1.1 ± 0.3	1.0 ± 0.3	1.1 ± 0.3	1.2 ± 0.4*,**	.049
LDL‐C (mmol/L)	2.3 ± 0.8	2.3 ± 0.8	2.3 ± 0.7	2.4 ± 0.8	.625
Gluocose (mmol/L)	5.6 (4.8, 7.2)	5.5 (4.8, 6.84)	5.6 (4.8, 7.0)	6.3 (5.0, 8.6)	.066
Echocardiography findings					
Time interval between echo (months)	9.137 ± 1.864	9.202 ± 1.846	9.119 ± 1.906	9.106 ± 1.775	.923
LVEF (%)	57.2 ± 2.6	57.1 ± 2.5	57.3 ± 2.5	56.6 ± 3.1	.131
LVPWT (mm)	10.3 ± 1.4	10.4 ± 1.4	10.1 ± 1.3*	10.7 ± 1.7**	.003
IVSD (mm)	10.9 ± 2.1	10.9 ± 2.1	10.7 ± 2.0	11.3 ± 2.4	.126
LAVI (ml/m^2^)	34.1 (25.7, 44.3)	34.4 (29.6, 41.3)	28.4 (21.4, 49.1)*	41.2 (36.2, 50.2)*,**	<.001
E/e'	10.9 (9.0, 14.9)	11 (9.0, 15.4)	10.3 (8.8, 14.1)	11.4 (9.0, 15.7)	.545

Abbreviations: ACEI, angiotensin‐converting enzyme inhibitor; ARB, angiotensin II receptor blocker; BMI, body mass index; BNP, B‐type natriuretic peptide; CCB, calcium channel blockers; HDL‐C, high‐density lipoprotein cholesterol; hs‐TnI, high‐sensitivity troponin; IVST, interventricular septal thickness; LDL‐C, low‐density lipoprotein cholesterol; LVEF, left ventricular ejection fraction; LVPWT, left ventricular posterior wall thickness; NT‐proBNP, N‐terminal pro B‐type natriuretic peptide; NYHA, New York Heart Association; PCI, percutaneous coronary intervention; TC, total cholesterol; TG, total triglyceride; UA, uric acid.

* *p* < .05 vs. deteriorated group

** *p* < .05 vs. stable group.

### 3.2 Predictors of LAVI

Univariate and multivariate linear logistic regression models were used to evaluate LAVI change in HFpEF patients. Variables independently associated with deteriorated LAVI included AF and higher creatinine (Supporting Information: Table 1), while variables predicting improved LAVI were the use of loop diuretics, CCB, and higher HDL‐C level (Supporting Information: Table 2).

### 3.3 Outcome analysis

**Figure 2:**
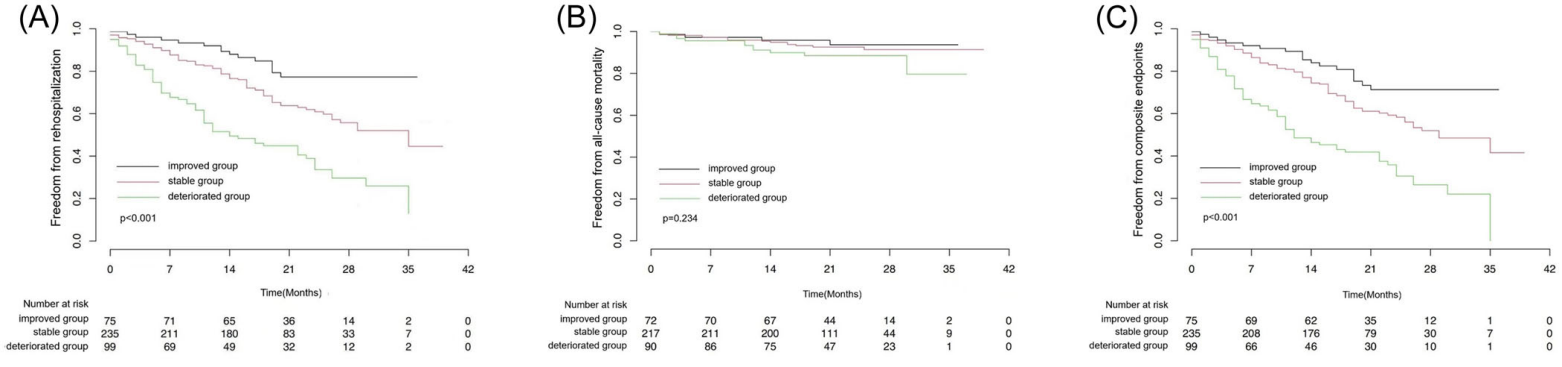
Kaplan–Meier curves for all‐cause rehospitalization (A) all‐cause mortality (B) and composite outcome (C) among three groups during follow‐up duration

During a median follow‐up of 22 months (interquartile range, 17–27 months), 182 participants (44.5%) experienced a composite outcome of rehospitalization or all‐cause death. Among them, 168 patients (41.1%) were rehospitalized and 31 patients (7.5%) died. The number of patients with rehospitalization was 65 (65.6%), 88 (37.4%), and 15 (20%), the mortality rates were 11 (11.1%), 16 (6.8%), and 4 (5.3%), and the percentage of patients with composite outcome were 68 (68.6%), 95 (40.4%), and 19 (25.3%) for the deteriorated, stable, and improved groups, respectively. As Kaplan–Meier curve showed (Figure 2), there was significantly difference of rehospitalization rate and composite outcome event rate among three groups (*p* < .001), whereas there was no statistical difference of mortality among them (*p* = .234).

The results of univariate and multivariate analyses were shown in Table 2. Compared with the improved group, the deteriorated group showed a significantly higher rate of rehospitalization (HR 4.548, *p* < .001) and a significantly higher composite outcome of rehospitalization or death (HR 3.868, *p* < .001) after univariate analysis. These associations persisted in the multivariate analysis (confounders adjusted for rehospitalization, all‐cause death, and composite outcome were shown in Supporting Information: Tables 3, 4, and 5), with adjusted HR 4.300, *p* < .001 for rehospitalization and adjusted HR 3.469, *p* < .001 for the composite outcome. The stable group was then compared to the improved group, and also showed higher HR for rehospitalization (HR 2.080, *p* = .005 for univariate analysis and HR 2.221, *p* = .005 for multivariate analysis) and higher HR for the composite outcome of rehospitalization or death (HR 1.751, *p* = .026 for univariate analysis and HR 1.830, *p* = .018 for multivariate analysis).

**Table 2:** Risk of death or rehospitalization in HFpEF subgroups

	Univariate analysis		Multivariate analysis	
Variables	Hazard Ratio (95% CI)	*p* Value	Hazard Ratio (95% CI)	*p* Value
Deteriorated vs. improved				
All‐cause rehospitalization	4.548 (2.592–7.978)	<.001	4.300 (2.438–7.586)	<.001
All‐cause death	2.326 (0.740–7.312)	.148	3.145 (0.818–12.093)	.095
All‐cause rehospitalization or all‐cause death	3.868 (2.326–6.433)	<.001	3.469 (2.071–5.812)	<.001
Stable vs. improved				
All‐cause rehospitalization	2.080 (1.203–3.596)	.005	2.221 (1.276–3.865)	.005
All‐cause death	1.316 (0.440–3.936)	.623	3.406 (0.922–12.579)	.066
All‐cause rehospitalization or all‐cause death	1.751 (1.070–2.865)	.026	1.830 (1.108–3.022)	.018

*Note*: Adjusted confounders were presented in Supporting Information: Tables 3, 4, and 5.

Abbreviations: CI, confidene interval; HFpEF, HF with preserved ejection fraction.

## 4 DISCUSSION

In the present study, we found change in LAVI was an independent predictor for rehospitalization in patients with HFpEF. Deteriorated LAVI was associated with worse clinical outcomes, whereas improved LAVI usually portended a favorable prognosis. Independent predictors for deteriorated LAVI included AF and higher creatinine level, whereas the use of loop diuretics, CCB, and higher HDL‐C level were associated with improved LAVI.

The diagnosis of HFpEF is challenging due to the normal ejection fraction. LV diastolic dysfunction is a common feature in HFpEF,^10^ and is crucial for the diagnosis of HFpEF.^5^ LV stiffness increases during ventricular diastole dysfunction, causing an increase in LA pressure to maintain adequate LV filling, ultimately leading to chamber dilatation and stretch of the atrial myocardium. Thus, the volumetric measurement of the LA by LAVI is an adequate analysis to predicate the LV filling pressures,^11^ providing a powerful predictive marker of LV diastolic dysfunction. LA volume is 66% larger in HFpEF patients compared with age‐matched control subjects,^12^ which is caused by multiple diseases, not only atrial diseases like AF,^13^ but also ventricular diseases including systemic hypertension, ischemic heart disease and hypertrophic cardiomyopathy, and valve diseases due to pressure or volume overload.^14^ In clinical practice, LAVI is routinely assessed by 2D echocardiography because of its portability, safety, and low cost. Currently, the prognostic impacts of LAVI are controversial in HFpEF.^15^, ^16^ A possible explanation is that LA volume dynamically changed over time. Most of LA remodeling is permanent, whereas others are reversible. Effective interventions such as angiotensin‐converting enzyme inhibitors (ACEIs) and angiotensin II receptor blockers (ARBs) or ablation for AF may arrest and perhaps reverse LA remodeling, with a consequence of reducing LA volume and improving atrial function.^17^, ^18^, ^19^

Nevertheless, little is known from published studies about the prognostic values of LAVI change in HFpEF. In our study, two echocardiography measurements showing change in LAVI was performed between 6 and 12 months. Moreover, we clearly demonstrated that deteriorated LAVI (permanent remodeling) was associated with negative prognostic impacts. As compared with patients had stable LAVI or improved LAVI, those with deteriorated LAVI had higher risk of rehospitalization and composite outcome after adjustment for potential confounders. In contrast, improved LAVI (reverse LA remodeling) was associated with improved prognosis in HFpEF. Indeed, deteriorated LAVI usually represented a state of maladaptive deterioration whereas improved LAVI reflected improvement in response to pharmacological or surgical/catheter interventions.

The prevalence of AF in HFpEF is relatively high, ranging from 15% to 40% across reports.^20^ Zakeri et al.^21^ found that 29% of AF was reported before HFpEF diagnosis, 23% concurrent with HFpEF, and 15% after diagnosis. In this cohort, the incidence of AF was approximately 55%. AF and HFpEF are often intricately linked through shared risk factors, such as advanced age, hypertension, obesity, smoking, coronary artery disease, and sleep apnea.^22^ Previous studies have demonstrated that cardioversion or catheter ablation for AF reduced the risk of AF recurrence and decreased LA volume.^23^, ^24^ Rhythm management of AF in HFpEF patients was associated with improved diastolic function and other endpoints.^25^, ^26^, ^27^ In the present study, we also found AF was an independent predictor for deteriorated LAVI. On the other hand, our results showed that taking loop diuretics and CCB, as well as higher HDL‐C levels predicted improved LAVI. In the latest ESC HF guideline,^5^ diuretics are recommended in congested patients with HFpEF to alleviate symptoms and signs (Class I, level C). Improving the state of congestion in the heart leads to a decrease in left ventricular end‐diastolic filling pressure, further reducing LA pressure and volume. CCB, an effective antihypertensive drug, is capable of decreasing cytoplasmic calcium concentration,^28^ and thus improving myocardial relaxation and reversing LA remodeling. Obesity and metabolic syndrome are common comorbidities in both AF and HFpEF patients.^29^ Derangement in HDL particle subfractions was found in HFpEF patients and was independently associated with adverse outcomes.^30^ Moreover, increased serum HDL‐C was recognized as a protective factor for LA remodeling.^29^ Although there is still lack of pharmacological interventions to improve the level of HDL‐C, circulating HDL‐C could be used as a predictor of disease progression in HFpEF patients.

In the current study, only 7.5% of HFpEF patients died over the approximately 2‐year of follow‐up, which was lower than reported elsewhere.^4^, ^31^ This discrepancy may be attribute to different inclusion and exclusion criteria of the cohort compared to the previous study. In this study, we excluded those with elderly aged ≥85 years, severe renal dysfunction, malignancy, and moderate or severe valve diseases, which would predispose the patients to higher rates of mortality. Unfortunately, in this study, the risk of mortality among three groups was not statistically different, but improved group showed a trend to reduce the rate of mortality (Stable vs. Improved HR 3.406, 95% CI 0.922–12.579, *p* = .066; Deteriorated vs. Improved HR 3.145, 95% CI 0.818–12.093, *p* = .095). The lack of association between LAVI change and mortality in HFpEF may be due to the small population.

## 5 LIMITATIONS

We recognized that there were several limitations in this current study. First, this was a small, retrospective, and single‐center study, which inevitably resulted in selection bias and recall bias. Second, the enrolled population was inpatients, and the study conclusions were not suitable for outpatients. Third, due to the lack of details of electrocardiogram, drug therapy and surgical operations for AF patients, we were incapable of investigating the associations between intervention strategies for AF and LAVI change. Fourth, the composition of stable group is complex, but we didn't perform a further detailed subgroup analysis.

## 6 CONCLUSION

In conclusion, the change in LAVI provides an important and dynamic morphophysiological marker of LV filling status that can be used to evaluate the disease progression of LV diastolic dysfunction in HFpEF patients. Increased LAVI represents a state of maladaptive deterioration whereas decreased LAVI reflects improvement in response to interventions. Aggressive risk factor management, especially AF control, may improve outcomes in HFpEF patients.

## CONFLICT OF INTEREST

The authors declare no conflict of interest.

## Supporting information

Supporting information.

## Data Availability

The data set that was used to support the results and conclusion of this study are included within the manuscript. The additional data are available upon reasonable request.
