# Supplementary material for: The predictive value of changes in left atrial volume index for rehospitalization in heart failure with preserved ejection fraction
Source: Clin Cardiol. 2022 Nov 20;46(2):151–8. doi: 10.1002/clc.23952 (PMC9933114; doi:10.1002/clc.23952)
Supplement: Supplementary file 1 — Supporting information. [file CLC-46-151-s001.doc]

**Supplemental table 1: Logistic Regression for deteriorated LVAI**

|  | Univariate analysis | | | Multivariate analysis | | |
| --- | --- | --- | --- | --- | --- | --- |
|  | OR | 95% CI | P value | OR | 95% CI | P value |
| Atrial fibrillation | 2.184 | 1.347-3.541 | 0.002 | 1.911 | 1.161-3.144 | 0.01 |
| Systolic blood  pressure | 0.990 | 0.980-1.000 | 0.041 | 0.990 | 0.980-1.000 | 0.08 |
| Creatinine | 1.004 | 1.000-1.008 | 0.030 | 1.005 | 1.000-1.009 | 0.013 |

Adjusted for atrial fibrillation, systolic blood pressure and creatinine.

**Supplemental table 2: Logistic Regression for improved LVAI**

|  | Univariate analysis | | | Multivariate analysis | | |
| --- | --- | --- | --- | --- | --- | --- |
|  | OR | 95% CI | P value | OR | 95% CI | P value |
| Hypertension | 1.885 | 1.038-3.426 | 0.037 | 1.160 | 0.570-2.359 | 0.683 |
| Loop Diuretics | 2.640 | 1.394-4.998 | 0.003 | 2.770 | 1.215-6.314 | 0.015 |
| β blocker | 2.342 | 1.076-5.098 | 0.032 | 2.062 | 0.909-4.675 | 0.083 |
| Spironolactone | 1.796 | 1.066-3.027 | 0.028 | 0.923 | 0.460-1.849 | 0.820 |
| CCB | 2.484 | 1.461-4.223 | 0.001 | 2.146 | 1.164-3.955 | 0.014 |
| HDL-C | 2.422 | 1.145-5.122 | 0.021 | 2.552 | 1.169-5.574 | 0.019 |
| LVPWT | 1.256 | 1.059-1.489 | 0.009 | 1.119 | 0.922-1.360 | 0.255 |

CCB, calcium channel blocker; HDL-C, high-density lipoprotein cholesterol; LVPWT, left ventricular posterior wall thickness. Adjusted for loop diuretics, β blocker, spironolactone, calcium channel blocker, high-density lipoprotein cholesterol and left ventricular posterior wall thickness.

**Supplemental table 3: Confounders adjusted for Rehospitalization**

|  | Univariate analysis | | | Multivariate analysis | | |
| --- | --- | --- | --- | --- | --- | --- |
|  | HR | 95% CI | P value | HR | 95% CI | P value |
| Diabetes | 1.413 | 1.038-1.925 | 0.028 | 1.168 | 0.840-1.623 | 0.356 |
| β blocker | 0.698 | 0.490-0.993 | 0.046 | 0.861 | 0.596-1.244 | 0.426 |
| Haemoglobin | 0.983 | 0.977-0.990 | <0.001 | 0.988 | 0.981-0.995 | 0.001 |
| Creatinine | 1.006 | 1.004-1.009 | <0.001 | 1.005 | 1.002-1.009 | 0.005 |
| UA | 1.001 | 1.00-1.002 | 0.041 | 1.000 | 0.999-1.001 | 0.590 |
| HDL-C | 0.413 | 0.249-0.684 | 0.001 | 0.598 | 0.354-1.009 | 0.054 |

HDL-C, high-density lipoprotein cholesterol; UA, uric acids. Adjusted for diabetes, β blocker, haemoglobin, creatinine, uric acids and high-density lipoprotein cholesterol.

**Supplemental table 4: Confounders adjusted for All-cause death**

|  | Univariate analysis | | | Multivariate analysis | | |
| --- | --- | --- | --- | --- | --- | --- |
|  | HR | 95% CI | P value | HR | 95% CI | P value |
| Loop Diuretics | 2.646 | 1.015-6.893 | 0.046 | 2.085 | 0.683-6.366 | 0.197 |
| Haemoglobin | 0.977 | 0.964-0.991 | 0.001 | 0.944 | 0.979-1.009 | 0.441 |
| BNP | 1.001 | 1.001-1.002 | <0.001 | 1.000 | 1.000-1.001 | 0.144 |
| Creatinine | 1.006 | 1.002-1.009 | 0.001 | 0.994 | 0.985-1.003 | 0.203 |
| UA | 1.004 | 1.002-1.006 | <0.001 | 1.001 | 0.999-1.004 | 0.264 |
| Urea | 1.132 | 1.094-1.171 | <0.001 | 1.159 | 1.079-1.246 | <0.001 |
| LVEF | 0.868 | 0.766-0.983 | 0.026 | 0.793 | 0.689-0.913 | 0.001 |

BNP, B-type natriuretic peptide; UA, uric acid; LVEF, left ventricular ejection fraction. Adjusted for loop diuretics, haemoglobin, B-type natriuretic peptide, creatinine, uric acid, urea and left ventricular ejection fraction.

**Supplemental table 5: Confounders adjusted for Rehospitalization or All-cause death**

|  | Univariate analysis | | | Multivariate analysis | | |
| --- | --- | --- | --- | --- | --- | --- |
|  | HR | 95% CI | P value | HR | 95% CI | P value |
| Diabetes | 1.39 | 1.032-1.870 | 0.03 | 1.092 | 0.785-1.519 | 0.603 |
| β blocker | 0.691 | 0.491-0.971 | 0.033 | 0.86 | 0.601-1.233 | 0.412 |
| Diastolic blood pressure | 1.012 | 1.001-1.023 | 0.035 | 1.016 | 1.004-1.027 | 0.007 |
| Haemoglobin | 0.983 | 0.977-0.989 | <0.001 | 0.989 | 0.982-0.997 | 0.004 |
| Creatinine | 1.007 | 1.005-1.009 | <0.001 | 1.003 | 0.999-1.007 | 0.110 |
| Urea | 1.06 | 1.044-1.076 | <0.001 | 1.020 | 0.987-1.054 | 0.242 |
| UA | 1.002 | 1.001-1.003 | <0.001 | 1.001 | 1.000-1.002 | 0.212 |
| HDL-C | 0.399 | 0.246-0.649 | <0.001 | 0.701 | 0.308-1.595 | 0.397 |

HDL-C, high-density lipoprotein cholesterol; UA, uric acids. Adjusted for diabetes, β blocker, diastolic blood pressure, haemoglobin, creatinine, urea, uric acids and high-density lipoprotein cholesterol.
